# The Diagnostic Relevance of β-D-Glucan for Candidemia within Internal Medicine Wards

**DOI:** 10.3390/diagnostics12092124

**Published:** 2022-09-01

**Authors:** Silvia Corcione, Luisa Chasseur, Tommaso Lupia, Nour Shbaklo, Silvia Scabini, Claudia Filippini, Simone Mornese Pinna, Stefania Morra di Celle, Rossana Cavallo, Francesco Giuseppe De Rosa

**Affiliations:** 1 Department of Medical Sciences, Infectious Diseases, University of Turin, 10126 Turin, Italy; 2 School of Medicine, Tufts University, Boston, MA 02111, USA; 3 Department of Medical Sciences, Internal Medicine, University of Turin, 10126 Turin, Italy; 4 Unit of Infectious Diseases, Cardinal Massaia, 14100 Asti, Italy; 5 Department of Anesthesia and Critical Care, University of Turin, 10126 Turin, Italy; 6 Microbiology and Virology Unit, University of Turin, 10126 Turin, Italy

**Keywords:** β-D-glucan, candidemia, internal medicine, yeast, fungi, biomarkers

## Abstract

Candidemia diagnosis is based on the combination of clinical, microbiological and laboratory data. We aimed to evaluate performances and accuracy of (1,3)-β-D-glucan (BDG) at various cut-offs in internal medicine patients. An observational retrospective–prospective study was performed. Patients with at least two determinations of BDG and paired, associated blood cultures within ±48 h were considered. A total of 140 patients were included: 26 with *Candida* spp. blood-stream infections (BSI) and 114 without candidemia. Patients with candidemia were older and had higher BDG values, need of parenteral nutrition, higher colonization by *Candida* in more than one site, presence of percutaneous gastrostomy and higher *Candida* or Charlson scores. BDG maintained the best compromise between sensitivity, specificity and optimal negative predictive value was 150 pg/mL. BDG values at cut-off of 150 pg/mL increase the strength of association between BDG and development of candidemia (Odds Ratio—OR 5.58; CI 2.48–12.53 vs. OR 1.06; CI 1.003–1.008). Analyzing BDG > 150 pg/mL along with Candida score > 2 and Charlson score > 4, the strength of the association amongst BDG, clinical scores and development of candidemia is increased. The overall clinical evaluation with the help of scores that consider BDG values > 150 pg/mL, Candida score > 2 and Charlson score > 4 in combination seems to predict better the need of antifungal empiric treatment.

## 1. Introduction

Candidemia is increasingly recognized as a frequent problem among patients hospitalized in internal medicine wards (IMWs), owing to the high prevalence of frail patients in this setting [1,2,3,4,5,6,7]. Such patients often have several risk factors for candidemia, including mucosal or cutaneous barrier disruptions, invasive procedures, endovascular devices, parenteral nutrition, malignancies, chronic renal failure, immunosuppressive treatments and extensive exposure to broad-spectrum antibiotics [6,8,9,10,11,12]. 

Moreover, mortality rates of candidemia recorded in IMWs are high; the rates are often comparable to those reported in intensive care units (ICUs) [3,4,7,13,14,15]. Despite attention to candidemia in medical wards, the evidence base for diagnosis and management is primarily derived from other settings, such as ICU, transplant units, surgery or in hematology wards, and most guidelines are directed towards these settings [16,17,18]. 

However, patients in IMW are usually more complex cases and often have more comorbidities than those traditionally studied in earlier studies on candidemia [1,2,3,4,5,6,7,16]. Measuring bloodstream infections remain the gold standard for diagnoses of candidemia; however, autoptic studies showed a sensitivity ranging from 27% to 71% [18].

(1,3)-β-D-glucan (BDG) testing is currently widely used as a surrogate marker to support the early diagnosis of candidemia [15,16,19]. Despite its diagnostic limitations, its high negative predictive value makes it an essential tool in antifungal stewardship programs to discontinue unnecessary empirical treatments [15,16,20]. 

Several studies have evaluated the performance of BDG in diagnosis, and they have shown that the sensitivity varies widely with a range of 47–100%, being attributed to differences in study design, patient populations, and commercial kits [19,20,21,22].

The primary objective of this study is to identify a cut-off value of BDG that is more reliable for candidemia in IMWs patients, using blood cultures positive for *Candida* spp. as a referral and diagnostic tool. 

## 2. Materials and Methods

A retrospective–prospective observational study was performed on patients hospitalized in medical wards (ordinary and sub-intensive units) at the City of Health and Science, Molinette Hospital, Turin, from January 2016 to December 2019. Patients with at least two determinations of BDG and associated blood cultures within ±48 h from their BDG samples were included. For all patient demographics, clinical and microbiological data were collected. 

Collected data about infections contained information on infection sites, etiology, and presence of sepsis or septic shock according to the Sepsis-3 criteria [23]. For each patient, data regarding antibiotic and antifungal therapy were reported. Specifically, the duration, dose, and combination therapy of each patient was collected in the data.

**Figure 1:**
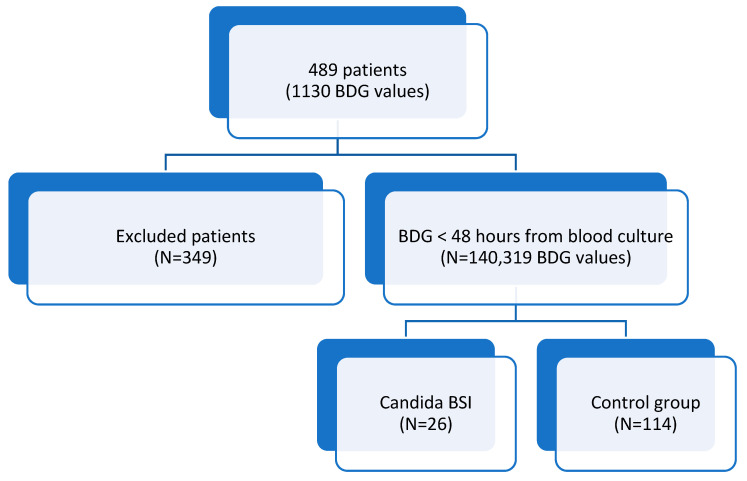
PRISMA flow diagram for included patients.

Considering patients with at least two BDG results, a total of 1130 BDG values corresponding to 489 patients were reported. BDG has a high negative predictive value (NPV), but it is well-known that several factors may have an impact on BDG values (Figure 1). 

Therefore, in order to have the best correlation between blood culture (considered gold standard for candidemia) and BDG, we decided to include only BDG performed together with blood culture to avoid isolated BDG positivity or negativity. Thus, 319 values were reported for 140 patients.

If the BDG was tested in more than one blood withdrawal, the earliest value was considered. If the BDG value was sourced from central and peripheral withdrawals, both results were considered. A database was then created to associate BDG values with clinical characteristics and biochemical values. The biochemical values considered were those carried out in the BDG testing. The BDG cut-off value that best represents the probability of candidemia was identified using the receiver operating characteristic (ROC) curve and analysis of sensitivity, specificity, positive predictive value (PPV), and NPV.

To describe clinical characteristics, the patient population was further divided into a *Candida* group (patients with blood cultures positive for *Candida* spp.) and compared to a second group, consisting of patients with blood cultures negative for *Candida* spp.

The demographic, epidemiological, and clinical characteristics of patients with Candida BSI were compared to the second group, consisting of patients with blood cultures negative for *Candida* spp. The data were expressed as mean ± standard deviation (SD), frequencies, or percentages with 95% confidence intervals (95% confidence interval—CI). The chi-square test was used to compare the distribution of categorical variables. Numeric variables that are normally distributed were analyzed by t-test, while variables that were not normally distributed were analyzed by Mann–Whitney test. To calculate the diagnostic performance of various BDG cut-offs, data were calculated for sensitivity, specificity, PPV, and NPV of the test (95% CI). Through the ROC curve, the best cut-off for candidemia was identified. The risk of having candidemia was assessed through logistic regression. The assessed independent variables were the BDG value (BDG > 150), percutaneous endoscopic gastrostomy (PEG), Candida score, and Charlson score. Age, parenteral nutrition, Candida colonization in more than one site, and dementia were not independently introduced into the regression since they were already included in the Candida and Charlson scores. Logistic regression analysis was performed on the entire population. Analysis was performed with SPSS^®^ version 20.0 (IBM^®^, Chicago, IL, USA). A *p*-value of 0.05 was used for significance. 

## 3. Results

Of these, 26 (18.6%) had positive blood cultures for Candida (Figure 1). One-hundred forty subjects were included in the study. There were 85 males (60.7%), and the average age was 63.3 ± 15.2 years. Subjects with candidemia more frequently had parenteral nutrition (*p* = 0.003), PEG (*p* = 0.043), and dementia (*p* = 0.002) (Table 1). They also had a known Candida colonization in more than one site (*p* = 0.043). Patients in the Candida BSI group had significantly higher Candida and Charlson scores (*p* = 0.002 and 0.037, respectively), whilst no statistical difference between groups was found for the qSOFA score (Table 1).

Regarding BDG values, the Candida BSI group had a significantly higher median BDG value compared to the second group (387 ± 187.6 pg/mL vs. 225 ± 193.3 pg/mL; *p* = 0.001). Clearly, patients in the Candida BSI group more frequently underwent echocardiography (*p* = 0.005) (Table 1).

Sensitivity, specificity, PPV, and NPV analysis of β-D-glucan were used for the diagnosis of candidemia in internal medicine. 

**Figure 2:**
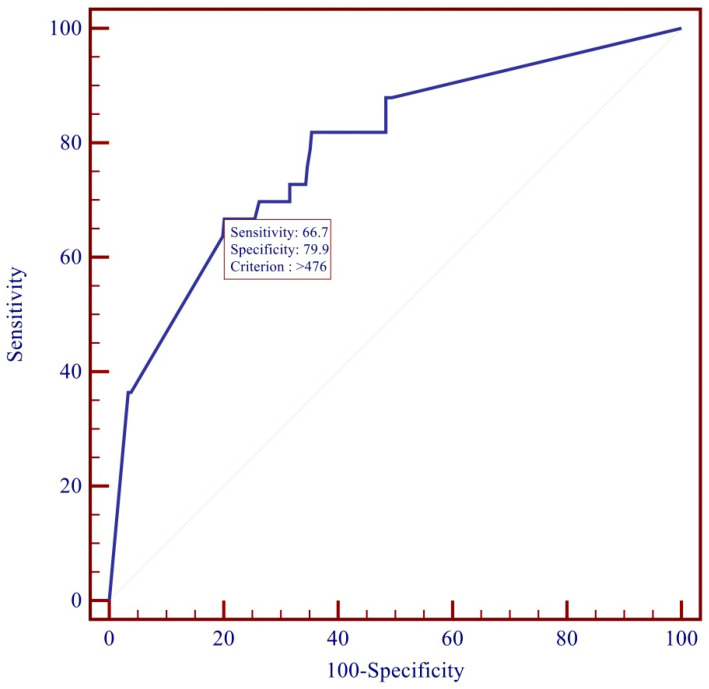
ROC curve for Beta-D-Glucan.

Through the ROC curve, applied to the whole study population, it was possible to identify the best cut-off for candidemia ascertained by Youden’s test. In our study, the best cut-off for the BDG was found to be 476 pg/mL. This value was associated with a sensitivity of 66% and a false-positive rate of 12%. The value also allows the maximization of the difference between true positives and false positives (Youden = 0.46 or 46%) for the identification of candidemia (Figure 2).

In our population, a value greater than 476 pg/mL was identified in 64% of the Candida BSI group. However, this value was associated with a sensitivity of 66% (Table 2). According to our analysis, the cut-off of 150 pg/mL was the value associated with a good sensitivity and specificity while maintaining an excellent NPV (AUC ROC 0.787) (Table 2). 

### Predictive Risk Factors for the Development of Candidemia

The multivariate analysis was performed on case and control groups (N = 140 patients). The risk of presenting Candida BSI was assessed through logistic regression analysis. In this analysis, the presence or absence of candidemia have been used as the dependent variable; in addition, the BDG value (at BDG > 150 pg/mL), PEG, Candida score, and Charlson score have been used as independent variables. 

The development of candidemia was found to be significantly associated with BDG value (*p* < 0.0001), Charlson score (*p* = 0.012) and Candida score (*p* = 0.022). The presence of PEG was protective (*p* = 0.001). However, the low number of patients with PEG (three in the Candida group and three in the second group) proved to be inadequate for statistical analysis (Table 3).

Using the BDG value at the cut-off of 150 pg/mL in the model of logistic regression, an increase in the strength of the association between BDG values, clinical scores, and developing candidemia (BDG: OR 4.24; CI 2.89–9.48; Candida score OR 3.24; CI 1.45–7.22; Charlson score OR 2.13; IC 0.99–4.56) was found (Table 3).

## 4. Discussion

Candidemia has been studied mainly in patients admitted to intensive care and onco-hematology units [20,21,22]. Few diagnostic tools are available so far to identify candidemia in internal medicine patients early, considering all the developed scores are related to ICU or hematological patients [16,17,18,19,20]. The recently released guidelines of the European Society of Clinical Microbiology and Infectious Diseases (ESCMID) also concern the non-ICU population [17]; this may be due to the fact that epidemiological data in internal medicine are probably underestimated [24]. 

The correct and early diagnosis of candidemia is highly unlikely because of vague symptoms, risk factors, and comorbidities [25]. Blood cultures still remain the gold standard for the diagnosis of candidemia; however, a lower sensitivity has been reported in the literature [19]. Therefore, BDG can be a useful tool to rule out candidemia in patients with a high risk for invasive candidiasis, thanks to its high negative predictive value [20]. 

The goal of our retrospective study was to evaluate the sensitivity, specificity, positive and negative predictive values, and accuracy of BDG at various cut-offs in internal medicine patients. The diagnostic accuracy of the serum BDG assay for the diagnosis of candidemia in this patient cohort was assessed by examining serological test results and blood culture results. The strength of this study lies in the diagnostic accuracy accomplished by considering only patients with at least two BDG tests within 48 h of BDG and blood testing. From our study, the value of BDG, which was the best compromise between sensitivity, specificity, and maintained negative predictive value, was 150 pg/mL (AUC ROC: 0.787 95% CI: 0.74–0.82; NPV of 95.7% (CI 95%: 93.22–98.18)). The risk factors that we reported for candidemia were consistent with similar studies: aging, NPE, PEG, Candida colonization in multiple sites, and high BDG values [26,27,28]. 

There are several limitations to this study. First, this is a single-center study that may not accurately reflect the general demographics of Italy. Furthermore, another limit of the study was the small sample that could not assess any potential evidence of BDG association with Candidemia in general. Nevertheless, within the limits of the study, it should be mentioned that candidemia was diagnosed through blood culture known to have a sensitivity of approximately 50%; our score cannot consider candidemia a with negative blood culture. Moreover, except for four cases reported on previous antifungal therapy (Table 1), we did not define candidemia for the whole population if the samples were drawn while already on antifungal treatment. No significant difference was found in the qSOFA score between the two groups. In contrast, Candida and Charlson scores were found to be higher in patients with candidemia. This result confirms the assertion that numerous comorbidities increase the risk for Candida BSI. After analyzing the ROC curves of BDG, the study found that the BDG value tends to predict candidemia more than clinical scores. These results confirm the importance of a joint evaluation between clinical and diagnostic tests. In particular, the study highlighted a BDG value greater than 150 pg/mL, a Candida score greater than 2, and a Charlson score greater than 4 for developing Candida BSI.

In conclusion, invasive Candida infections remain difficult to diagnose accurately, especially in non-hematological patients [1,2,3,4,5,6,7,29]. Analyzing blood cultures remains the diagnostic gold standard despite having a sensitivity of around 50%. The combination of the results of BDG and blood culture examinations is useful for ruling out candidemia and avoiding unnecessary treatment [1,2,3,4,5,6,7,29,30].

For this reason, this study was conducted to define the accuracy of BDG in non-hematological patients, considering the results of blood cultures as reference data as we stated also in the limits of the study. From the data obtained, the BDG cut-off value that revealed better diagnostic accuracy was 150 pg/mL. In clinical practice, the greatest utility of the test is represented by its high NPV, which allows for the discontinuation of antifungal therapy with a good safety margin. 

## 5. Conclusions

The overall clinical evaluation of a patient is essential with the help of scores considering risk factors. We support, with the limits of this study, that in the presence of BDG values > 150 pg/mL, Candida score > 2, and Charlson score > 4, it is appropriate to initiate empirical antifungal therapy even in the absence of positive blood cultures. Moreover, in the presence of BDG < 150 pg/mL and in the absence of certain risk factors, it is also possible to suspend antifungal therapy with a good safety margin.

## Figures and Tables

**Table 1:** Characteristics of the patients.

	Total Patients N = 140 (100)	Candida BSI N = 26 (18.5)	Control Group N = 114 (81.4)	*p*-Value
**Demographics**				
Age (years), *mean (SD)*	63.3 (15.2)	69.57 (16.8)	61.92 (14.5)	**0.009**
Sex (M, %)	85 (60.7)	17 (65.4)	68 (59.6)	0.549
**Hospital ward**				
Ordinary medicine, (%)	83 (59.3)	18 (69)	65 (57)	0.253
Subintensive medicine, (%)	57 (40.7)	8 (30.8)	49 (43)	0.253
**Risk factors for Candida Infection**				
CVC, (%)	114 (81.4)	23 (88.5)	91 (79.8)	0.307
Previous antibiotic use, (%)	62 (44.3)	10 (38.5)	52 (45.6)	0.495
Previous use of azoles, (%)	5 (3.6)	1 (3.8)	4 (3.5)	0.933
Concomitant antibiotic use, (%)	138 (98.6)	26 (100)	112 (98.2)	0.435
Parenteral nutrition, (%)	56 (40)	17 (65.4)	39 (34.2)	**0.003**
Chemotherapy, (%)	8 (5.7)	0 (0)	8 (7)	0.154
**Hospitalization>10 d in previous 3 months, (%)**	54 (38.6)	11 (42.3)	43 (37.7)	0.564
Candidemia in previous 3 months, (%)	4 (2.9)	0 (0)	4 (3.4)	0.333
Candida colonization in >1 site, (%)	6 (4.3)	3 (11.5)	3 (2.6)	**0.043**
Transferred from ICU, (%)	19 (13.6)	3 (11.5)	16 (14)	0.737
**Transferred from long term care, (%)**	9 (6.5)	3 (11.5)	6 (5.3)	0.830
Transferred from surgery ward, (%)	7 (5)	2 (7.7)	5 (4.4)	0.495
**Long term care in previous 3 month, (%)**	11 (7.9)	3 (11.5)	8 (7)	0.439
Dialysis, (%)	18 (12.9)	3 (11.5)	15 (13.2)	0.824
**Surgery in the 30 days before, (%)**	23 (16.4)	6 (23)	17 (14.9)	0.311
PEG, (%)	6 (4.3)	3 (11.5)	3 (2.6)	**0.043**
Pancreatitis, (%)	2 (1.4)	1 (3.8)	1 (0.9)	0.250
Abdominal surgery, (%)	23 (16.4)	6 (23.1)	17 (14.9)	0.311
Steroid therapy, (%)	61 (43.6)	9 (34)	52 (45)	0.307
Stoma, (%)	9 (6.5)	1 (3.8)	8 (7)	0.562
**Comorbidities**				
Diabetes, (%)	34 (24.3)	7 (26.9)	27 (23.7)	0.726
Solid organ cancer, (%)	25 (17.9)	5 (19.2)	20 (17.5)	0.839
Cirrhosis, (%)	21 (15)	5 (19.2)	16 (14)	0.254
Dementia, (%)	6 (4.3)	4 (15.4)	2 (1.8)	**0.002**
Moderate to severe CKD, (%)	19 (13.5)	4 (15.4)	15 (13.2)	0.984
*CDI*, (%)	16 (11.4)	5 (19.2)	11 (9.5)	0.168
Immunosuppressive therapy, (%)	20 (14.3)	3 (11.5)	17 (14.9)	0.857
Atrial fibrillation, (%)	20 (14.3)	2 (7.7)	18 (15.8)	0.287
Hypertension, (%)	73 (52.1)	16 (51.5)	57 (50)	0.288
COPD, (%)	18 (12.8)	4 (15.4)	14 (12.3)	0.670
CHF, (%)	17 (12.1)	3 (11.6)	14 (12.3)	0.917
CAD, (%)	20 (14.3)	2 (7.7)	18 (15.8)	0.287
Cerebral accident, (%)	17 (12.1)	3 (11.5)	14 (12.3)	0.917
PMN < 0.5 ×10^9^/L, (%)	8 (5.7)	1 (3.6)	7 (6.1)	0.649
**Imaging**				
Echocardiography, N (%)	84 (60)	22 (84.5)	62 (54)	**0.005**
**Laboratory tests**				
**BDG, *mean (SD), pg/mL***	203 (188)	387 (187.6)	225 (193.3)	**<0.001**
**WBC, *mean (SD), ×10^9^/L***	10.010 (6.255)	11.913 (6.820)	11.988 (6.973)	0.94
**Creatinine, *mean (SD), g/dL***	1.29 (1.21)	1.54 (1.38)	1.57 (1.44)	0.96
**eGFR, *mean (SD), mL/min***	97.2 (73.2)	105.9 (75.7)	100.3 (67.9)	0.83
**CRP, *mean (SD), mg/dL***	78.8 (75.17)	91.6 (71.2)	120 (111.8)	0.81
**PCT, *mean (SD), ng/mL***	3.4 (10.5)	3.2 (6.1)	5.6 (15.5)	0.6
**Albumin, *mean (SD), g/mL***	2.7 (0.5)	2.6 (0.5)	2.8 (0.5)	0.07
**Scores**				
qSOFA, *mean (SD)*		1.27 (0.724)	1.18 (0.868)	0.424
Candida score, *mean (SD)*		2.04 (1.183)	1.20 (1.130)	**0.002**
Charlson score, *mean (SD)*		5.77 (2.833)	4.49 (2.468)	**0.037**

**Abbreviations:** BSI: bloodstream infection; CVC: central venous catheter; ICU: intensive care unit; PEG: percutaneous endoscopic gastrostomy; CDI: Clostridium Difficile infection; CKD: chronic kidney disease; COPD: chronic obstructive pulmonary disease; CHF: chronic heart failure; CAD: coronary artery disease; PMN: polymorfonuclear; BDG: beta-D-glucan; WBC: white blood cell; eGFR: estimate glomerular filtration rate; CRP: c reactive protein; PCT: procalcitonin.

**Table 2:** Diagnostic performance of BDG at different cut-offs.

BDG Test	Values of BDG Cut-Off (pg/mL)									
	60		80		150		200		476	
	Estimate	95% CI	Estimate	95% CI	Estimate	95% CI	Estimate	95% CI	Estimate	95 % CI
**Sensitivity %**	87.8	71.8–96.6	81.8	64.5–96.0	81.8	64.5–93.0	69.7	51.3–84.4	66.6	48.2–82.0
**Specificity %**	50.6	45.6–55.7	54.96	49.9–60.0	64.5	59.2–68.9	69.4	64.7–74.0	79.9	75.6–83.7
**PPV (%)**	13.7	9.22–18.18	13.2	8.63–17.77	15.1	9.75–20.45	14.7	9.01–20.39	34.0	21.0–47.0
**NPV (%)**	96.5	93.97–99.03	95.8	93.14–98.46	95.7	93.22–98.18	94.3	91.59–97.01	90.0	84.0–96.0
**AUC ROC**	0.787	0.74–0.82	0.787	0.74–0.82	0.787	0.74–0.82	0.787	0.74–0.82	0.787	0.74–0.82

**Abbreviations:** BDG: beta-D-glucan; PPV: positive predictive value; NPV: negative predictive value; AUC: area under the curve; CI: confidence interval.

**Table 3:** Risk factors for candidemia in Internal Medicine Ward patients.

Parameter	OR	CI 95 %	*p*-Value		OR	CI 95 %	*p*-Value
**BDG**	1.006	1.003–1.008	<0.0001	**BDG > 150 pg/mL**	**5.585**	2.488–12.537	**<0.0001**
**PEG**	0.137	0.041–0.463	0.0014	**PEG**	0.188	0.059–0.600	**0.0047**
**Candida score**	1.466	1.056–2.035	0.0221	**Candida score**	1.411	1.037–1.919	**0.0283**
**Charlson score**	1.218	1.044–1.420	0.0121	**Charlson score**	1.153	1.012–1.313	**0.0321**

**Abbreviations:** BDG: beta d glucan; PEG: percutaneous endoscopic gastrostomy.

## Data Availability

Not applicable.
